# Temporal Patterns and Intra- and Inter-Cellular Variability in Carbon and Nitrogen Assimilation by the Unicellular Cyanobacterium *Cyanothece* sp. ATCC 51142

**DOI:** 10.3389/fmicb.2021.620915

**Published:** 2021-02-04

**Authors:** Lubos Polerecky, Takako Masuda, Meri Eichner, Sophie Rabouille, Marie Vancová, Michiel V. M. Kienhuis, Gabor Bernát, Jose Bonomi-Barufi, Douglas Andrew Campbell, Pascal Claquin, Jan Červený, Mario Giordano, Eva Kotabová, Jacco Kromkamp, Ana Teresa Lombardi, Martin Lukeš, Ondrej Prášil, Susanne Stephan, David Suggett, Tomas Zavřel, Kimberly H. Halsey

**Affiliations:** ^1^Department of Earth Sciences, Utrecht University, Utrecht, Netherlands; ^2^Institute of Microbiology, Czech Academy of Sciences, Centre Algatech, Třeboň, Czechia; ^3^Max Planck Institute for Marine Microbiology, Bremen, Germany; ^4^Sorbonne Université, CNRS, Laboratoire d’Océanographie de Villefranche, Villefranche-sur-mer, France; ^5^Sorbonne Université, CNRS, Laboratoire d’Océanographie Microbienne, Banyuls-sur-mer, France; ^6^Institute of Parasitology, Czech Academy of Sciences, Biology Centre, České Budějovice, Czechia; ^7^Centre for Ecological Research, Balaton Limnological Institute, Tihany, Hungary; ^8^Botany Department, Federal University of Santa Catarina, Campus de Trindade, Florianópolis, Brazil; ^9^Department of Biology, Mount Allison University, Sackville, NB, Canada; ^10^Laboratoire de Biologie des Organismes et Ecosystèmes Aquatiques, FRE 2030, Muséum National d’Histoire Naturelle, CNRS, IRD, Sorbonne Université, Université de Caen Normandie, Normandie Université, Esplanade de la Paix, France; ^11^Global Change Research Institute, Czech Academy of Sciences, Brno, Czechia; ^12^STU-UNIVPM Joint Algal Research Center, Marine Biology Institute, College of Sciences, Shantou University, Shantou, China; ^13^NIOZ Royal Netherlands Institute for Sea Research and Utrecht University, Den Burg, Netherlands; ^14^Universidade Federal de São Carlos, São Carlos, Brazil; ^15^Department Experimental Limnology, Leibniz-Institute of Freshwater Ecology and Inland Fisheries, Stechlin, Germany; ^16^Department of Ecology, Berlin Institute of Technology, Berlin, Germany; ^17^University of Technology Sydney, Climate Change Cluster, Faculty of Science, Ultimo, NSW, Australia; ^18^Department of Microbiology, Oregon State University, Corvallis, OR, United States

**Keywords:** *Crocosphaera subtropica* (former *Cyanothece* sp. ATCC 51142), *Cyanothece*, photosynthesis, carbon fixation, nitrogen fixation, nanoSIMS, TEM

## Abstract

Unicellular nitrogen fixing cyanobacteria (UCYN) are abundant members of phytoplankton communities in a wide range of marine environments, including those with rapidly changing nitrogen (N) concentrations. We hypothesized that differences in N availability (N_2_ vs. combined N) would cause UCYN to shift strategies of intracellular N and C allocation. We used transmission electron microscopy and nanoscale secondary ion mass spectrometry imaging to track assimilation and intracellular allocation of ^13^C-labeled CO_2_ and ^15^N-labeled N_2_ or NO_3_ at different periods across a diel cycle in *Cyanothece* sp. ATCC 51142. We present new ideas on interpreting these imaging data, including the influences of pre-incubation cellular C and N contents and turnover rates of inclusion bodies. Within cultures growing diazotrophically, distinct subpopulations were detected that fixed N_2_ at night or in the morning. Additional significant within-population heterogeneity was likely caused by differences in the relative amounts of N assimilated into cyanophycin from sources external and internal to the cells. Whether growing on N_2_ or NO_3_, cells prioritized cyanophycin synthesis when N assimilation rates were highest. N assimilation in cells growing on NO_3_ switched from cyanophycin synthesis to protein synthesis, suggesting that once a cyanophycin quota is met, it is bypassed in favor of protein synthesis. Growth on NO_3_ also revealed that at night, there is a very low level of CO_2_ assimilation into polysaccharides simultaneous with their catabolism for protein synthesis. This study revealed multiple, detailed mechanisms underlying C and N management in *Cyanothece* that facilitate its success in dynamic aquatic environments.

## Introduction

Nitrogen fixing microorganisms (diazotrophs) are critical suppliers of bioavailable forms of nitrogen (N, e.g., ammonium) in natural ecosystems. In the surface ocean where primary production is often limited by N availability, unicellular N_2_-fixing cyanobacteria (UCYN) are now recognized as having key roles in biogeochemical cycles (Zehr et al., 2001; Karl et al., 2002; Montoya et al., 2004; Zehr, 2011; Wilson et al., 2017).

Because of the scarcity of cultured representatives, *Cyanothece* sp. ATCC 51142 (henceforth *Cyanothece* 51142, recently reclassified as *Crocosphaera subtropica*; Mareš et al., 2019) has become an experimental model of UCYN (Reddy et al., 1993; Schneegurt et al., 1994; Colón-López and Sherman, 1998; Sherman et al., 1998; Li et al., 2001). The sequenced *Cyanothece* 51142 genome and controlled *Cyanothece* culture studies have provided insights into the genetic controls underlying the temporal segregation of N_2_ fixation activity, regulation of photosynthesis (Stöckel et al., 2008; Toepel et al., 2008; Welsh et al., 2008), and carbon (C) metabolism (Schneegurt et al., 1994; Colón-López and Sherman, 1998; Aryal et al., 2011; Bernstein et al., 2015). *Cyanothece* 51142 appear to restrict N_2_ fixation to the night time to protect the N_2_ fixing enzyme complex, nitrogenase, from inactivation by molecular oxygen produced by photosynthesis during the day time (Reddy et al., 1993; Welsh et al., 2008). Intracellular storage of the newly fixed C and N facilitates temporal separation of activities. Specifically, N fixed during the night is initially stored as cyanophycin until it is used for processes such as protein and nucleic acid synthesis, whereas C fixed during the day is stored as polysaccharides until it is respired the following night to supply reducing equivalents (NADPH) and ATP needed to support N_2_ fixation (Schneegurt et al., 1994; Li et al., 2001; Großkopf and LaRoche, 2012; Inomura et al., 2019). C respiration during the late afternoon and night also facilitates nitrogenase activity by depleting molecular oxygen that diffuses into the cell from the environment (Dron et al., 2012; Großkopf and LaRoche, 2012; Červený et al., 2013; Inomura et al., 2019).

In addition to their ability to fix N_2_, *Cyanothece* (and other UCYN) can assimilate various forms of combined nitrogen (e.g., NH_4_, NO_3_, urea, and amino acids; Mulholland et al., 2001; Holl and Montoya, 2005; Dekaezemacker and Bonnet, 2011; Masuda et al., 2013). Combined nitrogen generally down-regulates N_2_ fixation because its uptake and assimilation are energetically less costly than the processes supporting nitrogenase activity (Mulholland et al., 2001; Holl and Montoya, 2005; Eichner et al., 2014). This flexibility in nitrogen metabolism is considered to be one reason why *Cyanothece* thrives in a variety of marine environments with widely ranging nitrogen concentrations (Rippka, 1988; Short and Zehr, 2007; Webb et al., 2009; Bonnet et al., 2011). *Cyanothece* 51142 appears to efficiently manage C and N storage using multiple copies of genes encoding enzymes in polysaccharide metabolism and conserved gene clusters that coordinate intersecting pathways of C and N metabolism (Welsh et al., 2008; Zhang et al., 2018). For example, the ornithine-ammonia cycle (OAC) may facilitate efficient sequestration and remobilization of N (Zhang et al., 2018). Other N-rich compounds, including arginine, can be catabolized to recycle N within the cell (Flores et al., 2019; Burnat et al., 2019). CO_2_ and N_2_ fixation activities are also at least partly regulated according to the cell cycle demands that vary across the day–night cycle (Dron et al., 2013). For example, the N demands of nucleic acid synthesis and the C and energy requirements for new cell synthesis generally restrict cell division to hours when intracellular N reserves and photosynthetic rates are high (Dron et al., 2013; Červený et al., 2013). In contrast to N_2_ fixation, combined N assimilation and photosynthetic C assimilation should not require temporal separation, raising questions about how N and C allocations change depending on whether cells are growing on N_2_ or combined N.

In this study, we characterized C and N assimilation rates over a diel cycle in *Cyanothece* 51142 grown under obligate diazotrophic and non-diazotrophic conditions (with added NO_3_). Our approach combined nanoscale secondary ion mass spectrometry (nanoSIMS) and transmission electron microscopy (TEM) to track the assimilation of ^13^C-labeled inorganic C and ^15^N-labeled N_2_ or NO_3_ into individual cells and resolve their assimilation into polysaccharides, cyanophycin, and other inclusion bodies over the day–night cycle. We observed clear differences in N allocation patterns, but also unexpected within-population heterogeneity, including wide variation in labeling of storage inclusions and day-time N_2_ fixation. We discuss these observations and highlight how careful evaluation of these complex nanoSIMS data revealed key metabolic mechanisms underlying C and N management in *Cyanothece* 51142 that facilitate its success in dynamic aquatic environments.

## Materials and Methods

### Bioreactor and Semi-Continuous Cultures

*Cyanothece* 51142 cultures were maintained in 400- or 1,000-mL flat panel photobioreactors (FMT 150, Photon Systems Instruments, Brno, Czech Republic) at 28°C and 300 μmol photons m^–2^ s^–1^ with a 14 h:10 h light:dark cycle (14L:10D), with L0 at 07:30 and D0 at 21:30. The initial and final hours of each light cycle were set to follow a sinusoidal increase or decrease in light intensity, respectively. Triplicate cultures were grown in ASP2 medium (Provasoli et al., 1957; Van Baalen, 1962) either supplemented with 17 mM NO_3_ (“NO_3_ culture”) or prepared with no inorganic N added (“N_2_ culture”). Cultures were bubbled with ambient air (400 mL min^–1^) and maintained under turbidostat mode at OD_680_ of ∼0.5. Another set of triplicate cultures were grown in ∼300 mL glass tubes in ASP2 medium without NO_3_ amendment at 28°C under 300 μmol photons m^–2^ s^–1^ (14L:10D, same light regime as above) under semi-continuous, fed-batch mode (“SC-N_2_ culture”). The SC-N_2_ cultures were cultivated and maintained to ensure a “back-up” option in case the turbidostat (N_2_) cultures failed during the experiment. Because both culture conditions were stable throughout the experiment, we opportunistically sampled both cultures. The SC-N_2_ culture exhibited a wider range of phenotypic variability compared to the N_2_ culture that may be akin to some more dynamic natural environments. Therefore, we chose to include results for both diazotrophic cultures. All cultures maintained constant population sizes for >21 days prior to sampling. Culture and cell characteristics for each treatment are given in Table 1. An analysis of photosynthetic electron transport and the energetic costs of N and C acquisition in the same cultures studied here are given in Rabouille et al. (In Revision).

**TABLE 1:** Culture conditions and properties of *Cyanothece* 51142 during N_2_ and NO_3_ growth.

Culture	Collection time	N_2_	NO_3_	SC-N_2_
Culturing strategy		Turbidostat	Turbidostat	Semi-continuous batch
Specific growth rate (d^–1^)^a^	Daily average	0.22 ± 0.07	0.31 ± 0.05	0.26 ± 0.09
Cell diameter (μm)	Dawn	2.92 ± 0.07	3.03 ± 0.09	2.99 ± 0.13
Chl *a* (fg cell^–1^)	Dawn	109 ± 14	144 ± 9	111 ± 9
Cell C (fg cell^–1^)	Dawn	2375 ± 23	2515 ± 159	3076 ± 127
Cell C (fmol cell^–1^)	Dawn	198 ± 23	209 ± 13	256 ± 13
Cell N (fg cell^–1^)	Dawn	534 ± 69	584 ± 30	550 ± 1.4
Cell N (fmol cell^–1^)	Dawn	38 ± 5	42 ± 2	39 ± 1
C:N (w:w)	Dawn	4.45 ± 0.58	4.30 ± 0.35	5.60 ± 0.22
C:N (mol:mol)	Dawn	5.19 ± 0.08	5.02 ± 0.08	6.53 ± 0.26
Polysaccharide content (fg glucose eq. cell^–1^)	Dawn	1657 ± 702	1978 ± 976	1166 ± 454^b^
Polysaccharides (fmol C cell^–1^)		55 ± 23	66 ± 33	39 ± 15^b^
Cyanophycin content (fg arginine eq. cell^–1^)	Light phase^c^	37 ± 22	81 ± 32	n.d.
Cyanophycin (fmol C cell^–1^)		2.1 ± 1.2	4.6 ± 1.8	–
Cyanophycin (fmol N cell^–1^)		1.1 ± 0.6	2.3 ± 0.9	–

*Shown are mean ± SD values for three replicate cultures.^a^ Determined by exponential fits of the OD_720_ signal from the on-board sensor in the turbidostats (Zavřel et al., 2015a) or by total volume displaced during daily dilution of semi-continuous batch culture.^b^ Measured at 16:00.^c^ Mean and SD calculated from three values measured 2, 7, and 14 h after the start of the light phase.*

Cell density and size distributions were determined using a Multisizer 4 Coulter Particle Counter (Beckman Coulter Inc., Brea, CA, United States). Particulate organic carbon (POC) and nitrogen (PON) were analyzed using an elemental analyzer (PerkinElmer PE2400, PerkinElmer Inc., Waltham, MA, United States) following sample collection (10 mL), centrifugation (28°C, 8,000 rpm, 7 min), and drying at 60°C. Chl *a* and polysaccharide contents were determined following the previously reported protocols (Zavřel et al., 2015a, b, 2018). Cyanophycin cell content was quantified by Sakaguchi reaction (Messineo, 1966), after sample concentration (30 mL) by centrifugation (28°C, 8,000 rpm, 7 min).

### Stable Isotope Probing Experiments

For stable isotope probing experiments, samples were collected from one culture replicate from each condition. Incubations were performed by sub-sampling cultures into 6 mL gas-tight vials and amending the ASP2 medium with NaH^13^CO_3_ (all cultures) and either ^15^N_2_ (N_2_ and SC-N_2_ cultures) or Na^15^NO_3_ (NO_3_ culture). Vials were incubated under light and temperature conditions that were equivalent to culture conditions. Incubation durations were 2 h in the morning, 2.5 h during the day, and 10 h during the night, with shorter incubations in early night (3 h) and late night (5 h). Isotope labeling was calculated from the known amounts of label added to the incubation medium and measured initial concentrations of unlabeled substrate in the bioreactors (Supplementary Table 1). The ^15^N_2_ enriched stock was prepared by injecting 10 mL of ^15^N_2_ gas into 43 mL of the ASP2 medium, followed by an equilibration for >24 h. The ^15^N-N_2_ atom fraction in the incubation medium was calculated assuming that ^15^N_2_ was fully equilibrated with the stock solution. Since this may lead to an underestimation of N_2_ fixation rates (Mohr et al., 2010), we refrain from comparisons of N assimilation rates between N treatments (N_2_ vs. NO_3_). However, comparisons over time and among cells within each treatment are not affected since any potential underestimation would be similar in all ^15^N_2_ incubations. NO_3_ concentration in the incubation medium was estimated by averaging NO_3_ concentrations measured in the bioreactor on the respective day of the experiment. Dissolved inorganic carbon (DIC) concentration in the incubation medium was estimated by measuring DIC concentrations in the bioreactor at three time points during the day and interpolating them to the starting time points of our stable isotope incubations. Because the DIC concentration in the cultures varied depending on the time of the day, ^13^C-DIC atom fractions varied during our incubations, although the amounts of added NaH^13^CO_3_ were the same (Supplementary Table 1).

### TEM Analysis

At the end of each isotope-labeling incubation, cells were collected and centrifuged at 2,700 rpm for 10 min at room temperature. One microliter of the pellet was mixed with 1 μL of 20% bovine serum albumin and transferred to a formvar-coated 100 mesh TEM grid. After removing the excess liquid with a filter paper, the grids were frozen in liquid ethane cooled with liquid nitrogen. Freeze-substitution was carried out in a 2% mixture of OsO_4_ in 100% acetone (v/v) sequentially at three temperatures: −90°C (for 96 h), −20°C (for 24 h), and 4°C (for 10 h). Temperature was increased at a rate of 5°C h^–1^ (from −90 to −20°C) and 3°C h^–1^ (from −20 to 4°C). After freeze-substitution, the samples were washed three times in acetone and infiltrated sequentially in a 2:1, 1:1, and 1:2 (v/v) mixture of acetone and low-viscosity Spurr resin (EMS) for 1 h in each step. Finally, the samples were incubated overnight in a 100% resin, transferred to embedding molds, and allowed to polymerize. Thin sections (200 nm) were cut with a diamond knife, placed on Cu-indexed TEM grids (rinsed in 30% ethanol), and contrasted for 20 min in saturated ethanolic uranyl acetate (EMS, Hatfield, United States; concentration 13 g/100 mL 50% ethanol; solution filtered before use through a 0.45 μm pore size filter). Images were taken using a JEOL 1010 TEM at 80 kV.

### NanoSIMS Analysis

Nanoscale secondary ion mass spectrometry analyses were performed on two types of samples: (i) thin sections that were first imaged by TEM (as described above) and (ii) whole cells collected on polycarbonate filters. For downstream analysis of samples initially imaged by TEM, the lowest primary ion beam current (0.5 pA) was used to achieve the highest lateral resolution afforded by the instrument (∼50 nm). However, because the samples were very thin (∼200 nm), the number of imaged frames was rather low (20–50) before the cell material was sputtered away. This sometimes resulted in a poor signal-to-noise ratio (SNR) in the final secondary ion images, and thus, a low number of cells for which good quality complementary TEM and nanoSIMS images are available. Additional measurements were therefore performed on cells deposited on filters, because the imaging could be done with a stronger beam (2 pA) and over a larger area and many more frames (>200). However, the improved throughput and SNR came at the expense of a lower spatial resolution (see section “Results”). For nanoSIMS analysis of whole cells, the cells were filtered onto polycarbonate filters (2.5 cm diameter, 0.2 μm pore size, Millipore), washed three times, air-dried, and stored at room temperature. Chemical fixation was not performed thus avoiding dilution of the isotope label. Just prior to nanoSIMS analysis, filters were sputter-coated with a 10-nm gold layer, cut into small circular pieces (5 mm diameter) suitable for the nanoSIMS sample holder, and imaged with a Neoscope II JCM-6000 scanning electron microscope (JEOL, Japan) to check sample quality (cell integrity and cell density).

Nanoscale secondary ion mass spectrometry measurements were performed with the NanoSIMS 50L instrument (Cameca, France) operated at Utrecht University. Areas of interest were first pre-sputtered with Cs^+^-ions until secondary ion yields stabilized. Subsequently, the primary Cs^+^-ion beam was scanned over the sample (areas between 10 μm × 10 μm and 30 μm × 30 μm in size, dwell time of 1 ms pixel^–1^) while detecting secondary ions ^12^C^–^, ^13^C^–^, ^16^O^–^, ^12^C^14^N^–^, ^12^C^15^N^–^, ^31^P^–^, and ^32^S^–^. To increase the overall signal, the same area was imaged multiple times, and the resulting ion count images were aligned and accumulated.

### NanoSIMS Data Processing and Quantification of Rates

Nanoscale secondary ion mass spectrometry data were processed with the Look@NanoSIMS software (Polerecky et al., 2012) to quantify ^13^C and ^15^N atom fractions, denoted as x(^13^C) and x(^15^N) (Coplen, 2011), in regions of interest (ROI’s) corresponding to cells or inclusion bodies (polysaccharide granules or cyanophycin inclusions). After drawing ROIs manually, *x*(^13^C) in the ROI was determined from the total counts of secondary ions ^12^C^–^ and ^13^C^–^ accumulated over the ROI pixels as *x*(^13^C) = ^13^C^–^/(^12^C^–^ + ^13^C^–^). Similarly, *x*(^15^N) in the ROI was determined from the total counts of ^12^C^15^N^–^ and ^12^C^14^N^–^ accumulated over the ROI pixels as *x*(^15^N) = ^12^C^14^N^–^/(^12^C^14^N^–^ + ^12^C^15^N^–^).

The C- and N-specific rates of ^13^C and ^15^N assimilation into whole cells (*k*_*C*_ and *k*_*N*_, respectively) were calculated as:

(1) kC=-1tln⁡[1-x(C13)-x(C13)inix(C13)S-x(C13)ini]

(2) kN=-1tln⁡[1-x(N15)-x(N15)inix(N15)S-x(N15)ini]

while the C- and N-specific rates of ^13^C and ^15^N incorporation into polysaccharide granules (*p*_*C*_ or *p*_*N*_, respectively) and cyanophycin inclusions (*y*_*C*_ and *y*_*N*_, respectively) were calculated as:

(3) pCoryC=1tx(C13)-x(C13)inix(C13)S-x(C13)ini

(4) pNoryN=1tx(N15)-x(N15)inix(N15)S-x(N15)ini

In Eqs 1–4, *x*(^13^C)_*S*_ and *x*(^15^N)_*S*_ are atom fractions of the C and N source, respectively, and *x*(^13^C)_*ini*_ and *x*(^15^N)_*ini*_ are the initial atom fractions of C and N in the ROI, respectively. The isotope labeling of the C and N sources, *x*(^13^C)_*S*_ and *x*(^15^N)_*S*_, was assumed to be constant during the incubation and was calculated as described above (Supplementary Table 1). *x*(^13^C)_*ini*_ and *x*(^15^N)_*ini*_ were determined by averaging data obtained from cells that were not exposed to the labeled substrate (i.e., control cells; *x*(^13^C)_*ini*_ = 1.052 × 10^–2^, SD(^13^C)_*ini*_ = 0.007 × 10^–2^, and *x*(^15^N)_*ini*_ = 3.75 × 10^–3^, SD(^15^N)_*ini*_ = 0.04 × 10^–3^, *n* = 30). A cell or an inclusion body was considered significantly enriched in ^13^C if the 95% confidence interval of its estimated mean ^13^C atom fraction did not overlap with that of the control cells, i.e., if *x*(^13^C) ± 2 × SE(^13^C) did not overlap with *x*(^13^C)_*ini*_ ± 2 × SE(^13^C)_*ini*_. Here, the standard errors were calculated as SE(^13^C)_*ini*_ = SD(^13^C)_*ini*_/$\sqrt{n}$ for the control cells, and SE(^13^C) = *x*(^13^C) × PE(^13^C) for each individual cell or inclusion body, where the relative Poisson error was calculated from the total counts of ^12^C^–^ and ^13^C^–^ in the cell or inclusion body as PE(^13^C) = [1 – *x*(^13^C)] × [1/^13^C^–^ + 1/^12^C^–^]^1/2^ (Polerecky et al., 2012). The same approach but using the total counts of ^12^C^14^N^–^ and ^12^C^15^N^–^ was applied to determine significant enrichment in ^15^N.

Note that the C- and N-specific rates of ^13^C and ^15^N assimilation into whole cells, polysaccharide granules, and cyanophycin inclusions have units of per time (i.e., h^–1^ or day^–1^) and give the rate of ^13^C and ^15^N assimilation rates *normalized* to the C and N content of the ROI [i.e., mol C (mol C)^–1^ h^–1^ for *k*_*C*_, *p*_*C*_, and *y*_*C*_, and mol N (mol N)^–1^ h^–1^ for *k*_*N*_, *p*_*N*_, and *y*_*N*_]. Evaluation of the variability in assimilation rates among cells and intracellular inclusions required considering how the measured *x*(^13^C) and *x*(^15^N) in the ROI were linked to cell growth and metabolism of internal C and N pools (e.g., synthesis and degradation of polysaccharides and cyanophycin inclusions, or recycling of N from existing proteins during cyanophycin synthesis). These considerations are summarized in the Discussion section (Section “Interpreting Isotopic Enrichment Imaging Data”). The assumptions underlying the rate calculations presented above are further explored and discussed in Polerecky et al. (In Revision).

## Results

### Identification of Intracellular Inclusions

**FIGURE 1:**
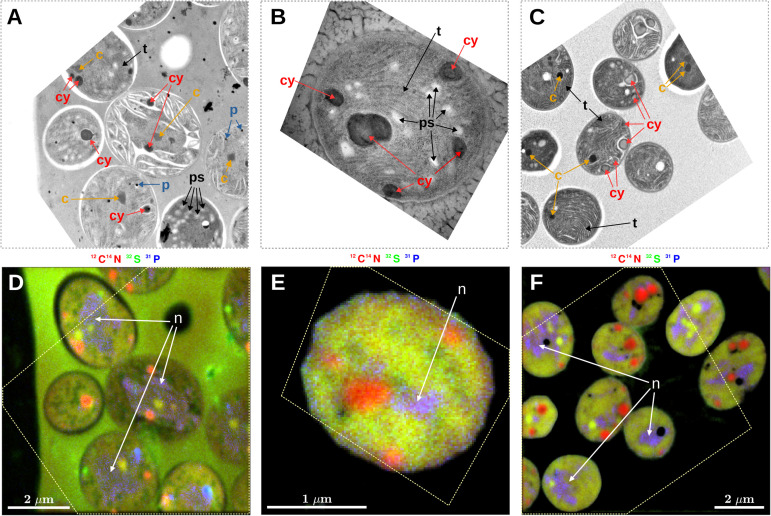
Correlative microscopy of thin sections of *Cyanothece* 51142 cells. Shown are examples of TEM images **(A–C)** and the corresponding nanoSIMS images **(D–F)**. The dashed polygon in panels **(D–F)** shows the boundary of the TEM image aligned within the NanoSIMS image. Shown are images from samples collected at 09:30 following a morning incubation (07:30–09:30) from the N_2_ culture **(A,D)** and NO_3_ culture **(B,E)**, and from a sample collected at 07:00 following a night-time incubation (21:45–07:00) from the NO_3_ culture **(C,F)**. Arrows in the images point to sub-cellular structures identified as carboxysomes (c), cyanophycin inclusions (cy), polyphosphate bodies (p), polysaccharide granules (ps), thylakoid membranes (t), and nucleoid (n). NanoSIMS images are shown as RGB overlays of secondary ion counts ^12^C^14^N^–^ (red), ^32^S^–^ (green), and ^31^P^–^ (blue). Note that the hues are not comparable among the images because, for each overlay, the contrast for the three color channels was modified so as to enhance the overall visibility of the intracellular variability. In addition to P^–^, the polyphosphate bodies had markedly increased O^–^ ion counts (data not shown).

Prominent intracellular inclusion bodies identified in TEM images of *Cyanothece* 51142 included carboxysomes, cyanophycin inclusions, polyphosphate bodies, polysaccharide granules, and thylakoid membranes (Figure 1). Some, but not all, of these inclusions could be reliably identified in nanoSIMS images when the accumulated secondary ion counts ^12^C^14^N^–^, ^32^S^–^, and ^31^P^–^ were combined into RGB overlays. Specifically, carboxysomes, which were identified in TEM images as dark areas with a characteristic hexagonal shape (Figures 1A,C, arrows labeled “c”), had relatively higher CN^–^ and S^–^ but lower P^–^ counts than the surrounding cell material. These differences caused carboxysomes to appear yellow-green in the RGB overlays (Figures 1D,F). Cyanophycin inclusions, which were identified through their oval shape and darker appearance in the TEM images, had markedly higher CN^–^ counts, while the S^–^ and P^–^ counts were not different from the surrounding cell material. The relative enrichment in CN^–^ counts gave cyanophycin inclusions an orange-to-red appearance in the RGB overlays (Figure 1, arrows labeled “cy”). Polyphosphate bodies had higher P^–^ counts and lower CN^–^ and S^–^ counts relative to the surrounding cell material and appeared as bluish spots in the RGB overlays (Figure 1, arrows labeled “p”). Although polysaccharide granules could be identified in TEM images as bright oval shapes (Figure 1A, arrows labeled “ps”; see also Deschamps et al., 2008), their identification from the nanoSIMS images was not reliable. For example, most polysaccharide granules were associated with localized decreases in CN^–^ counts (Figure 1D). However, the contrast between the polysaccharide granules and the cell matrix was low, and similar decreases in CN^–^ counts sometimes occurred even when there was no obvious presence of polysaccharide in the TEM images. Neither could variability in S^–^, P^–^, C^–^, or O^–^ counts (C^–^ and O^–^ data not shown) be used to distinguish polysaccharide granules in the nanoSIMS images. Similarly, thylakoid membranes were not identifiable using the nanoSIMS images although their visibility in the TEM images was often good (Figures 1A–C, arrows labeled “t”). Conversely, nucleoids were clearly observed as the violet-colored regions in the RGB overlays due to P counts being markedly higher than the surrounding cell material and CN^–^ and S^–^ counts that were similar to the surrounding cell material (Figures 1D–F, arrows labeled “n”); however, direct nucleoid identification in the TEM images was not possible.

### Carbon and Nitrogen Assimilation Rates and Allocation Patterns

#### N_2_ Culture

**FIGURE 2:**
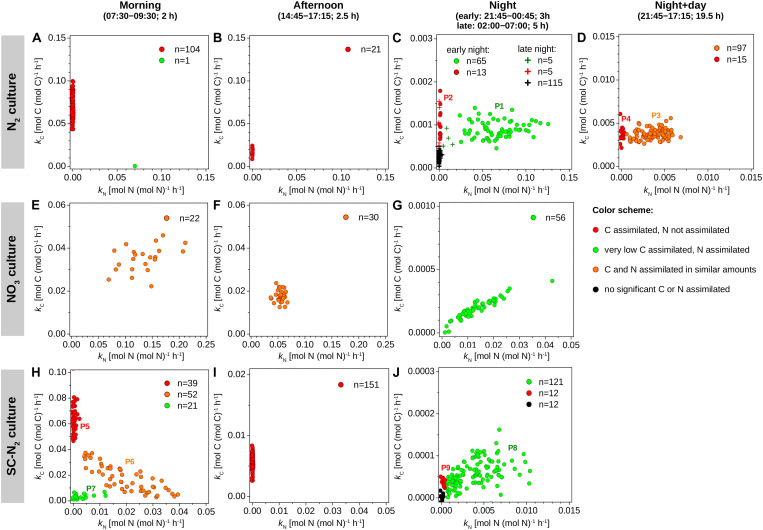
Element-specific rates of C and N assimilation in *Cyanothece* 51142. Data points in panels **(A–J)** show element-specific assimilation rates of ^13^C [*k*_*C*_, in mol C (mol C)^–1^ h^–1^] and ^15^N [*k*_*N*_, in mol N (mol N)^–1^ h^–1^] for individual cells from three parallel cultures (N_2_, diazotrophic culture grown in turbidostat mode; NO_3_, culture grown on nitrate in turbidostat mode; SC-N_2_, diazotrophic culture grown in semi-continuous batch mode). Cells were incubated during time intervals indicated above the graphs. L0 was 07:30 and D0 was 21:30. Data points are colored according to the amounts of C and N assimilated (see color scheme). The number of measured cells in each subpopulation is given in the legend. Subpopulations labeled P1–P9 are described in further detail in text. Note different scaling of the x and y axes among the different panels.

**FIGURE 3:**
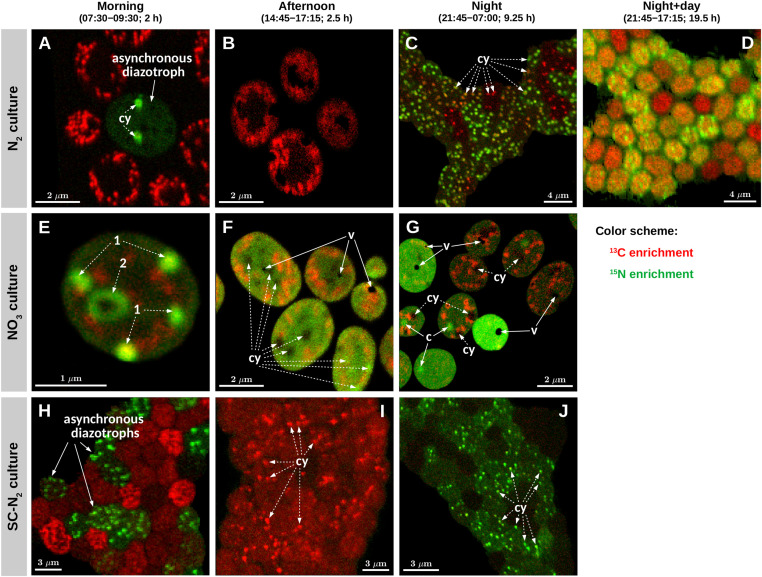
Images of isotopic enrichment in *Cyanothece* 51142. Shown are overlays of ^13^C (red) and ^15^N (green) enrichments measured in cells from three parallel cultures (N_2_, diazotrophic culture grown in turbidostat mode; NO_3_, culture grown on nitrate in turbidostat mode; SC-N_2_, diazotrophic culture grown in semi-continuous batch mode). Cells were incubated during time intervals indicated above the images. Images shown in panels **(A**,**B,E–G)** were obtained from thin cell sections analyzed by TEM [panels **(A,E,G)** correspond to panels **(A–C)** in Figure 1], whereas panels **(C,D,H–J)** show images of cells collected on polycarbonate membrane filters. Additional images of filtered cells are shown in Supplementary Figure 2. In each image, the intensity (“brightness”) of the red and green color scales linearly with the ^13^C and ^15^N enrichment, respectively, with black indicating no enrichment. Note, however, that because the scaling for the red and green colors was optimized independently for each image to enhance the visibility of the intracellular heterogeneity, the intensities of the red and green colors are not comparable among the images. In panels **(A,C,E–G,I,J)**, examples of cyanophycin inclusions (cy) and carboxysomes (c) are marked with dashed-line and solid-line arrows, respectively. In panels **(A,B,E–G)**, areas and spots with pronounced ^13^C enrichment (red) correspond to polysaccharide granules. Black areas in panels **(F,G)** correspond to voids (v) due to artifacts associated with the preparation of the thin cell sections.

Daytime C fixation in *Cyanothece* 51142 grown under diazotrophic conditions in turbidostat mode (“N_2_ culture”) was observed in all but one of the 126 cells imaged (Figures 2A,B). The C-specific rates of ^13^C assimilation in whole cells, *k*_*C*_, were highest in the morning and declined on average by about 80% in the afternoon (Table 2). ^13^C enrichment was highest in polysaccharide granules and lower and diffusely spread throughout the cell matrix (Figures 3A,B). The C-specific rates of ^13^C assimilation in polysaccharide granules, *p*_*C*_, varied (CV≈32%), with 61% of the variance explained by differences among cells and 39% of the variance explained by differences within cells (Supplementary Figure 1A). Moreover, the relative area of the cell sections covered by polysaccharide granules varied among cells (range: 0.08–0.28, CV≈43%) and was significantly positively correlated with the *k*_*C*_ values (*R* = 0.68, *p* = 0.002; Supplementary Figure 2B).

**TABLE 2:** ^13^C and ^15^N assimilation rates in *Cyanothece* during N_2_ and NO_3_ growth.

	Morning	Afternoon	Night		Night + day
	(07:30–09:30)	(14:45–17:15)	(early 21:45–00:45)	(late: 02:00–07:00)	(21:45–17:15)
***k*_*C*_ (d**^–^**^1^)**					
N_2_ culture	1.70 ± 0.35	0.39 ± 0.08	0.022 ± 0.006	0.007 ± 0.005	0.090 ± 0.015
NO_3_ culture	0.84 ± 0.15	0.42 ± 0.07	0.0045 ± 0.0019		–
SC-N_2_ culture	0.74 ± 0.63	0.14 ± 0.03	0.0012 ± 0.0008		–
***k*_*N*_ (d**^–^**^1^)**					
N_2_ culture	0.016 ± 0.164	0.0024 ± 0.0032	1.34 ± 0.79	0.012 ± 0.047	0.078 ± 0.044
NO_3_ culture	3.19 ± 0.91	1.31 ± 0.18	0.32 ± 0.18		–
SC-N_2_ culture	0.24 ± 0.28	0.0005 ± 0.0034	0.077 ± 0.063		–

*Shown are mean ± SD values of k_C_ and k_N_ for cells measured by nanoSIMS. Values for individual cells are shown in Figure 2.*

The majority of cells in the N_2_ culture fixed N_2_ during the night (Figure 2C), with the exception of one cell (out of 104 imaged) that fixed N_2_ in the morning (green circle in Figure 2A; “asynchronous diazotrophic” cell in Figure 3A). During the early night, cells grouped into two clear subpopulations exhibiting different activities: P1 showed significant N_2_ fixation and accounted for 83% of cells, while P2 showed no significant N_2_ fixation and accounted for 17% of cells (compare green and red circles in Figure 2C). P1 and P2 showed low but significant C fixation during the early night incubation (Figure 2C). The *k*_*C*_ values in P1 and P2 did not differ at night [ANOVA, F(1,77) = 1.64, *p* = 0.204] and were about 1–1.5% of morning *k*_*C*_ values. The *k*_*C*_ and *k*_*N*_ values did not correlate in subpopulation P1 (*R* = 0.043, *p* = 0.73), and *k*_*C*_:*k*_*N*_ ranged from 0.008 to 0.035 (mean = 0.016, SD = 0.008, CV≈50%) among the cells. During the late night, only about 3% of cells fixed N_2_ (green pluses in Figure 2C), and the average *k*_*N*_ was about 1% of the average *k*_*N*_ in the early night (Table 2). Significant C fixation was observed in 5% of cells, and these cells again grouped into subpopulations depending on whether they also fixed N_2_ (3%) or not (2%) (compare green and red pluses in Figure 2C). The remaining 95% of cells showed no detectable C nor N_2_ fixation during the late night (black pluses in Figure 2C).

The C and N_2_ fixation patterns observed separately during the day and night were reflected in cells incubated with isotopes over the full night + day cycle (Figures 2D, 3D). The majority of cells (∼87%) fixed both C and N_2_ (P3, orange circles in Figure 2D), whereas the remaining 13% of cells had *k*_*C*_ values similar to P3 but showed no significant N_2_ fixation (P4, red circles in Figure 2D). Average *k*_*C*_ and *k*_*N*_ values were similar for the majority of cells incubated for the full night + day cycle, but these values were only about 5% of the peak *k*_*C*_ and *k*_*N*_ values observed during the morning and early night, respectively (Table 2). These dramatic shifts in metabolism over the day caused *k*_*C*_ in the morning to overestimate specific growth rate (μ = 0.22 day^–1^) by 7.7-fold. Theoretically, the average night + day *k*_*C*_ value should estimate μ, but was only 0.09 day^–1^. Similarly, *k*_*N*_ at night overestimated μ by up to 6-fold, and the average night + day *k*_*N*_ was only 0.08 day^–1^. The discrepancies between μ and the average *k*_*C*_ and *k*_*N*_ from the night + day (∼20 h) incubations were likely caused by differences in incubation conditions, including gas flow and medium exchange in the turbidostat that were not possible in the isotope labeling incubation. Finally, *k*_*C*_:*k*_*N*_ varied widely among cells (range 0.6–3.3, mean = 1.24, SD = 0.78, CV = 63%).

**FIGURE 4:**
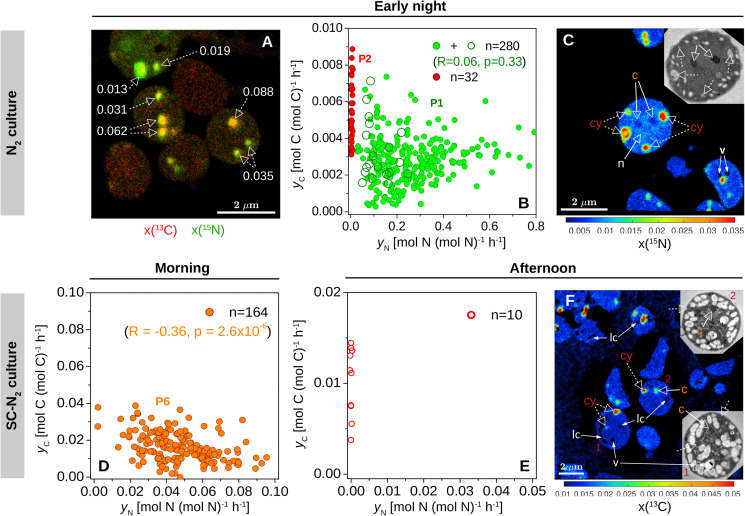
Within-cell heterogeneity of N assimilation in N_2_-fixing *Cyanothece* 51142. Shown are data for cells from the N_2_ culture (diazotrophic culture grown in turbidostat mode) incubated during early night [21:45–00:45; **(A–C)**] and from the SC-N_2_ (diazotrophic culture grown in semi-continuous batch mode) incubated during morning [7:30–9:30; **(D)**] and afternoon [14:45–17:15; **(E,F)**]. L0 was 07:30 and D0 was 21:30. Images show an overlay of the ^13^C (red) and ^15^N (green) atom fractions **(A)**, the ^15^N atom fraction **(C)**, and the ^13^C atom fraction **(F)**. The corresponding TEM images of selected cells are shown in the inset. Dashed-line arrows point to cyanophycin inclusions (cy), while solid-line arrows point to carboxysomes (c), voids due to artifacts associated with the preparation of the cell sections (v), and areas where the atom fractions could not be quantified due to low secondary ion counts (lc). In panel A, numbers indicate the ratios of C and N-specific rates of ^13^C and ^15^N assimilation in individual cyanophycin granules, *y*_*C*_:*y*_*N*_. In panel C, the ^15^N atom fractions in the marked inclusion bodies range between 0.022 and 0.035 for cyanophycin (cy), 0.0047 and 0.0052 for carboxysomes (c), and 0.0082 and 0.0083 for nucleoid (n), and are all significantly greater than in control cells [x(^15^N)_*ini*_ = 0.00375]. **(B,D,E)** Scatter plots of *y*_*C*_ vs. *y*_*N*_ in cyanophycin granules. Values depicted with open and filled symbols were derived from images obtained from thin cell sections and whole cells deposited on a filter, respectively. Data points are colored as in Figure 2. Correlation coefficients (*R*) and the corresponding *p*-values are also shown for green symbols in panel **(B)** (P1, corresponding to values from the P1 subpopulation shown in Figure 2C) and orange symbols in panel **(D)** (P6, corresponding to values from the P6 subpopulation shown in Figure 2H). Red data points correspond to cyanophycin inclusions with no significant ^15^N enrichment.

Cyanophycin inclusions showed the greatest ^13^C and ^15^N enrichment compared to other inclusions within individual cells from the early night incubation (Figures 3C, 4A–C). Similar to the data clustering observed for the whole cells, individual cyanophycin inclusions grouped into two clear subpopulations, one with significant ^15^N enrichment (P1) and one with no significant ^15^N enrichment (P2) (compare green and red circles in Figure 4B). There was no significant correlation between the C- and N-specific ^13^C and ^15^N assimilation rates in cyanophycin granules, *y*_*C*_ and *y*_*N*_, in P1 (*R* = 0.10, *p* = 0.10), and *y*_*C*_:*y*_*N*_ were highly variable between cyanophycin granules among cells (range: 0–0.09, mean = 0.015, SD = 0.016, CV = 107%) and within individual cells (Figure 4A). In one cell where we could clearly resolve all relevant intracellular structures, we observed low but significant ^15^N enrichment in the carboxysomes and a slightly greater ^15^N enrichment in the nucleoid in addition to the strong ^15^N enrichment in the cyanophycin inclusions (Figure 4C).

#### NO_3_ Culture

*Cyanothece* 51142 grown under non-diazotrophic conditions in turbidostat mode (with NO_3_ added; “NO_3_ culture”) showed daily patterns of C fixation that were similar to the N_2_ culture. Morning *k*_*C*_ was 2.7-fold greater than μ of 0.31 day^–1^. In the afternoon, *k*_*C*_ decreased on average by 50% and reached about 0.5% of the morning values during the night time (Figures 2E–G and Table 2). Cells in the NO_3_ culture always assimilated newly fixed C into polysaccharide granules (Figures 3E–G), whereas in the N_2_ culture the highest ^13^C enrichment was observed in polysaccharide granules during the day but in cyanophycin inclusions during the night (compare Figures 3A–C).

The daily patterns of N assimilation and intracellular allocation were more complex in the NO_3_ culture than in the N_2_ culture. Values of *k*_*N*_ were highest in the morning (10.3-fold greater than μ) and decreased by about 60% in the afternoon and by 90% during the night (Figures 2E–G and Table 2). In the morning, ^15^N was often accumulated in what appear to be newly synthesized cyanophycin inclusions (Figure 3E and Supplementary Figure 3D, arrows 1) or was added to existing cyanophycin inclusions as manifested by a ^15^N-rich “shell” surrounding a ^15^N-poor core (Figure 3E, arrow 2). In many cells, ^15^N enrichment was clearly present in the cell matrix but not in existing cyanophycin inclusions (Supplementary Figure 3D, arrows 3). In the afternoon, ^15^N enrichment was distributed relatively homogeneously within the cell matrix, and ^15^N enrichment in cyanophycin inclusions was sometimes greater but more often lower than in the cell matrix (Figures 3E,F). In the morning, *k*_*C*_ and *k*_*N*_ were significantly correlated (*R* = 0.43, *p* = 0.045), whereas no significant correlation was observed in the afternoon (*R* = −0.02, *p* = 0.90). For both morning and afternoon incubations, *k*_*C*_:*k*_*N*_ varied among individual cells from 0.15 to 0.5 (mean = 0.31, SD = 0.08, CV = 26%).

Night-time NO_3_ assimilation resulted in ^15^N being homogeneously enriched within the cell matrix, but carboxysomes were notably more enriched in ^15^N than the cell matrix, and cyanophycin inclusions showed no ^15^N enrichment (Figure 3G). This intracellular N allocation pattern was observed in all cells (Supplementary Figure 3F) despite the large intercellular variability in *k*_*N*_ values. In contrast to the N_2_ culture, *k*_*C*_ and *k*_*N*_ in the NO_3_ culture were strongly correlated during the night (*R* = 0.94, *p* < 10^–4^; Figure 2G) even though the newly assimilated ^13^C and ^15^N were allocated into different cell compartments (Figure 3G). Night-time *k*_*C*_:*k*_*N*_ values varied only slightly in the NO_3_ culture among individual cells (range 0.01–0.03, mean = 0.015, SD = 0.005, CV = 33%).

#### SC-N_2_ Culture

In the majority of *Cyanothece* 51142 cells grown under diazotrophic conditions in semi-continuous, fed-batch mode (“SC-N_2_ culture”), temporal patterns in *k*_*C*_ and *k*_*N*_ values as well as in C and N allocation were similar to the N_2_ culture grown in turbidostat mode. In these cells, the highest *k*_*C*_ and *k*_*N*_ values were observed during the morning and night, respectively (Figures 2H–J and Table 2). As in the N_2_ culture, the highest ^13^C and ^15^N enrichments were detected in the polysaccharide granules and cyanophycin inclusions, respectively (Figures 3H–J). In the afternoon, there was no measurable N_2_ fixation, and *k*_*C*_ values decreased by about 80% compared to the morning values (Figure 2I).

There were, however, two notable differences in the behaviors of the N_2_ (turbidostat) and SC-N_2_ cultures. Firstly, in the SC-N_2_ culture there was a large subpopulation of cells (∼46%) that fixed N_2_ during the morning (subpopulation P6 in Figure 2H). In these asynchronous diazotrophic cells, ^13^C and ^15^N enrichments were concentrated in cyanophycin inclusions (Figure 3H), and *k*_*N*_ and *k*_*C*_ as well as *y*_*C*_ and *y*_*N*_ were significantly negatively correlated (*R* = −0.81, *p* < 10^–4^, Figure 2H; *R* = −0.36, *p* < 10^–5^, Figure 4D, respectively). Additionally, the average *k*_*N*_ measured in these morning N_2_-fixing cells was 6.4-fold higher than the average *k*_*N*_ during the night time. Consequently, the average *k*_*N*_ for the SC-N_2_ culture in the morning was about 3.1-fold higher than at night (Table 2). The second notable difference was that cells from the SC-N_2_ culture incubated in the afternoon had some cyanophycin inclusions that were significantly more enriched in ^13^C compared to the polysaccharide granules and cell matrix (Figures 3I, 4F).

### Intercellular Heterogeneity

**FIGURE 5:**
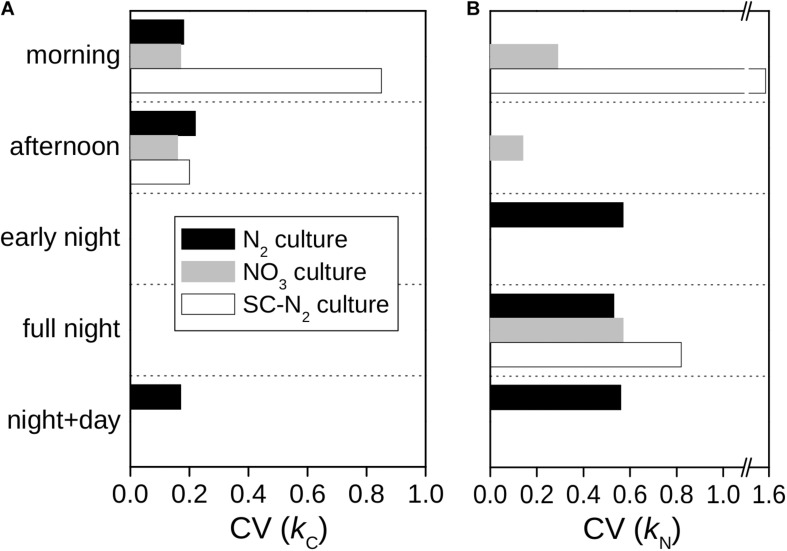
Intercellular variability of C and N assimilation rates in *Cyanothece* 51142. Shown are the coefficients of variation (CV) of C-specific **(A)** and N-specific **(B)** rates rates of ^13^C and ^15^N assimilation for whole cells, *k*_*C*_ and *k*_*N*_, derived from data shown in Figure 2.

In all cultures and incubations, *k*_*C*_ and *k*_*N*_ were markedly heterogeneous among individual cells (Figure 2). Intercellular heterogeneity in day-time *k*_*C*_ values was similar with CV = 16–22% (Figure 5) across the turbidostat cultures (N_2_ and NO_3_ cultures). Heterogeneity in night-time *k*_*N*_ values was also similar between the turbidostat cultures but was about 3-fold greater (CV = 53–57%) than heterogeneity in day-time *k*_*C*_ values. Heterogeneity in day-time *k*_*N*_ in NO_3_ cultures decreased from the morning to the afternoon (CV declined from ∼30 to 15%). Moreover, heterogeneity in *k*_*N*_ in the N_2_ culture during the night time was higher than in the NO_3_ culture during the morning.

The semi-continuous diazotrophic batch culture (SC-N_2_) showed considerably greater intercellular heterogeneity than the turbidostat cultures (N_2_ and NO_3_), especially for rates of N_2_ fixation (Figure 5). In the SC-N_2_ culture, the differentiation of cells into subpopulations in the morning was reflected in the high heterogeneity in *k*_*C*_ (CV = 85%) and *k*_*N*_ (CV = 155%). In the afternoon, heterogeneity in *k*_*C*_ in the SC-N_2_ culture decreased to a level similar to the N_2_ culture (CV = 20%).

## Discussion

### Interpreting Isotopic Enrichment Imaging Data

The data yielded from nanoSIMS analyses hold valuable information about metabolic strategies used by cells across time and space. However, knowledge about cells’ activities that influence their isotopic composition at the end of an SIP incubation is critical to properly interpret nanoSIMS data. Carbon and nitrogen assimilation in *Cyanothece* mainly occurred during short and intensive periods either in the few hours after dawn or during the night. These periods of rapid assimilation activities fueled the majority of the cells’ C and N needs for growth and were followed by long periods of very low assimilation rates. Our results also show a wide range of C- and N-specific rates of ^13^C and ^15^N assimilation within and between whole cells and among inclusions. Here, we critically evaluate the factors that can lead to variations in these measured rates and discuss several important, and to our knowledge previously unrecognized, considerations for using the spatially resolved ^13^C and ^15^N enrichment data obtained by nanoSIMS to infer rates of substrate assimilation. We first focus on principles of labeling as applied to subcellular structures, such as inclusion bodies, and then discuss these principles in the context of whole cell assimilation rates. A more comprehensive and mathematical analysis of these considerations can be found in Polerecky et al. (In Revision).

^13^C or ^15^N enrichment depends upon the amount of labeled C or N added to a structure during an incubation relative to the amount of unlabeled C or N present prior to the incubation. Any structure that is newly synthesized during an incubation will have ^13^C and ^15^N enrichments that match those of the enriched C and N sources. However, C or N that is added to an existing structure during the incubation will cause the average ^13^C and ^15^N enrichment measured in the structure to be lower than in the C and N sources. The deviation between structure enrichment and source enrichment will decrease with incubation time as a function of the rate of biosynthesis but increase with the initial C and N content of the structure. Consequently, variation in the initial C and N content of structures will lead to *apparent* differences in ^13^C and ^15^N enrichments among structures present in an incubation even though the rates of biosynthesis and accumulation of ^13^C and ^15^N may have been the same.

^13^C or ^15^N enrichment of a structure will also vary if the C and N used for its biosynthesis are derived from unlabeled sources of C and N, including the turnover of cellular macromolecules, in addition to the labeled sources external to the cell. One way to detect the relative importance of internal macromolecular recycling is to quantify the *ratio* of C- and N-specific rates of ^13^C and ^15^N incorporation into a structure (e.g., *y*_*C*_:*y*_*N*_ for a cyanophycin granule). Because the C:N ratio of many compounds comprising cell structures is well defined (e.g., cyanophycin has a C:N ratio of two), their biosynthesis will preserve the ^13^C:^15^N signature of the C and N sources (except for the minute deviations linked to kinetic isotope fractionation). Consequently, if only the external, labeled pools of C and N are utilized for biosynthesis, the ratio of the C- and N-specific rates of ^13^C and ^15^N assimilation must be equal to 1. Any departure of this ratio from 1 implies that some of the C or N in the structure originated from a different source (i.e., with a different ^13^C:^15^N signature than that of the externally supplied sources).

Similar reasoning is needed when analyzing the isotopic enrichment of whole cells. Average ^13^C or ^15^N enrichment of a cell depends upon the amount of labeled C or N taken up during an incubation relative to the amount of unlabeled C or N present in the cell prior to the incubation. Consequently, intercellular variability in the amounts of C and N storage compounds will lead to *apparent* differences in the cellular ^13^C and ^15^N enrichments among cells even if the rates of ^13^C and ^15^N assimilation into cells were same. The influence of varying storage compound content can be revealed by analyzing the ratio between the C- and N-specific rates of ^13^C and ^15^N assimilation into whole cells, *k*_*C*_:*k*_*N*_. For an individual cell, this ratio will be 1 (or very close to 1, if the subtle effects of kinetic isotope fractionation are included) provided the cell is in balanced growth, and the externally supplied labeled pools of C and N were the only sources of C and N assimilated by the cell. Any deviation from 1 indicates that (1) the cell assimilated an additional, unlabeled external source; (2) a storage product was preferentially synthesized over another (e.g., polysaccharides over cyanophycin); or (3) the cell recycled internal, unlabeled stores of C or N.

The foregoing analysis of enrichment sources, biosynthesis, and initial C and N content highlights that *k*_*C*_:*k*_*N*_ can reveal the presence, synthesis, or mobilization of intracellular C and N stores. With these ideas in mind, we evaluate the isotope enrichment results for *Cyanothece* 51142 cultures grown with different N sources across a day–night cycle to understand their C and N assimilation processes and allocation strategies.

### Roles of Internal C and N Recycling in Cyanophycin Synthesis

The majority of C used for cyanophycin synthesis at night in cells growing diazotrophically originated from recycling of existing C compounds within the cells. Cyanophycin synthesis involved some ^13^C (external C source) assimilation, but the *k*_*C*_:*k*_*N*_ and *y*_*C*_:*y*_*N*_ values were much lower than 1 (Figures 2C, 4A,B). Thus, the bulk of the CO_2_ incorporated into cyanophycin was likely derived from polysaccharide catabolism needed to simultaneously provide energy and reducing power (ATP and NADPH) for N_2_-fixation. Nevertheless, cyanophycin synthesis was detected via their enrichment in ^13^C rather than ^15^N in about 17% of cells (Figure 3C). ^13^C is assimilated via two CO_2_ fixation steps leading to synthesis of the non-ribosomal peptide, cyanophycin, which is comprised of aspartate and arginine (Flores et al., 2019). Specifically, CO_2_ is incorporated via (i) pyruvate carboxylase or phosphoenolpyruvate carboxykinase yielding oxaloacetate which is transaminated by glutamate to form aspartate and (ii) carbamoyl phosphate synthase together with ornithine transcarbamoylase operating to generate arginine (Zhang et al., 2018).

The differences in *y*_*C*_:*y*_*N*_ values between and within N_2_-fixing cells in the same culture (Figures 3C, 4A,B) were also caused by variations in the relative contributions to cyanophycin synthesis of ^15^N assimilated during the incubation and unlabeled N assimilated prior to the incubation. Unlabeled N may originate from efficient recycling of N in polyamines, including degradation of arginine via the recently described AgrE/PutA pathway (Burnat et al., 2019; Lee and Rhee, 2020). We hypothesize that the enrichment patterns observed in the N_2_ cultures at night were caused by a variable fraction of cyanophycin-N that originated from active N_2_ fixation (P1) or from protein degradation (P2). These findings suggest that diazotrophy demands internal N redistribution at night in all cells (as indicated by the similar ^13^C enrichment in cyanophycin inclusions), but distinct subpopulations emerge depending on their rates of N_2_-fixation.

Some cyanophycin inclusions in cells from the SC-N_2_ culture showed pronounced ^13^C but no ^15^N labeling during the afternoon incubation (Figures 3I, 4E,F). We speculate that these cells are part of the asynchronous diazotrophic morning subpopulation that fixed N_2_ into cyanophycin (P6, Figure 3H) but that later synthesized cyanophycin using internal (and unlabeled) N sources. This idea is supported by the observation that about half of the population fixed N_2_ in the morning and exhibited pronounced ^13^C enrichment in cyanophycin granules (Figures 3H,I). These observations suggest that cyanophycin synthesis in *Cyanothece* can occur throughout the entire light period, with cyanophycin-N derived either from N_2_-fixation or internal N (e.g., via protein degradation). Our high-resolution imaging shows that cyanophycin effectively collects, stores, and redistributes N to facilitate ongoing protein synthesis and catabolism.

### Cyanophycin Biosynthesis Is Prioritized, but N Can Flow Directly to Protein During Growth on NO_3_

Whether growing diazotrophically or with NO_3_, *Cyanothece* prioritized cyanophycin synthesis when the rates of N assimilation were at their highest. When N assimilation rates were lower, most cells growing on NO_3_ assimilated N into the cell matrix and carboxysomes, suggesting that N was used directly for protein synthesis without prior storage in cyanophycin. Night-time NO_3_ uptake into carboxysomes indicates that this new N was used immediately for the synthesis of RubisCO to maintain its content throughout the diel cycle (Nassoury et al., 2001). How cyanophycin synthesis is prioritized over protein synthesis is not clear, but our data suggest that once the cell has acquired sufficient N storage into cyanophycin, this storage step can be bypassed in favor of direct incorporation into proteins.

Night-time assimilation of N in the NO_3_ culture was accompanied by small but detectable assimilation of CO_2_, indicative of pyruvate carboxylase activity during the night. Surprisingly, this new C was directed into polysaccharides (Figure 3G). Typically, the pyruvate carboxylase reaction is considered important to ensure availability of oxaloacetate for citrate synthase to initiate the TCA cycle that produces NADH and amino acid precursors. However, in NO_3_-grown cells, existing polysaccharides appear to have supplied all of the C for protein synthesis (because no ^13^C was incorporated into the carboxysomes coincident with ^15^N; see above). These results suggest that the amphibolic nature of the glycolytic/gluconeogenic pathway is directional with respect to the flow of newly fixed C into polysaccharides: newly fixed C flows through gluconeogenesis into polysaccharides simultaneous to glycolytic catabolism of “old C” stored in polysaccharides for use in protein synthesis. Glycolytic and gluconeogenesis pathways are strictly controlled so that they cannot be both highly active at the same time, which would create a futile cycle. The highly sensitive detection of labeled C and N afforded by the stable isotope probing and nanoSIMS technologies combined with TEM allowed us to view these unexpected cell activities that occur at very low levels but that support the careful modulation of C and N storage and re-mobilization in *Cyanothece.*

### Intercellular Heterogeneity in C and N Metabolism

Within-population heterogeneity in ^13^C and ^15^N enrichments (*k*_*C*_ and *k*_*N*_), such as that observed in N_2_ and SC-N_2_ treatments, has been reported in previous nanoSIMS-based studies (Foster et al., 2013; Mohr et al., 2013; Masuda et al., 2020), although the causes remain poorly understood (Ackermann, 2015). Intercellular heterogeneity has been attributed to stochastic gene expression or state switching in fast growing bacteria and yeast (Elowitz et al., 2002; Blake et al., 2003; Raj and van Oudenaarden, 2008; Raser and O’Shea, 2013; Sanchez et al., 2013; Damodaran et al., 2015). *Cyanothece* metabolism is strongly regulated by circadian rhythms, and thus, the intercellular heterogeneity observed in our study is at least partly associated with the regulation of C and N fixation determined by the light period and cell cycle (Caudron and Barral, 2013; Bach and Taucher, 2019).

We find that the metabolism of internal C and N storage compounds is another mechanism contributing to cell-to-cell heterogeneity in isotopic enrichment. Dual-label stable isotope probing combined with sub-cellular resolution imaging enabled us to identify internal recycling of N during cyanophycin synthesis, which led to variation in *k*_*N*_ values during night-time N_2_ fixation (CV≈55%; Figure 2C) and to the wide ranges of *y*_*N*_ (CV≈72%; Figure 4B) and *y*_*C*_:*y*_*N*_ (Figures 4A,B) values.

The intercellular heterogeneity in *k*_*C*_ values during morning C fixation could be caused by the variable polysaccharide content among cells (see Section “Interpreting Isotopic Enrichment Imaging Data”). The limited cellular volume probed by the nanoSIMS measurement also likely contributes to an apparent population heterogeneity. These alternatives are supported by the variable content and uneven distribution of polysaccharide granules within cells (Figures 1, 3A,B,E–G) and the fact that a large fraction of the variability in *k*_*C*_ values (∼50%) was explained by the areal coverage by polysaccharide granules (Supplementary Figure 1B).

Differences in the turnover rates of storage inclusions with different C:N contents (cyanophycin vs. polysaccharides) may underlie the three-fold mismatch between the cell-to-cell variation in *k*_*N*_ and *k*_*C*_. Other cyanobacteria and pico-eukaryotes have exhibited similar differences in *k*_*N*_ and *k*_*C*_ (Berthelot et al., 2019; Masuda et al., 2020). One explanation is that day-time acquisition of C reserves was insufficient to fuel N-fixation and other night-time metabolisms (Dron et al., 2013). However, pre-dawn cells were never completely depleted of polysaccharide granules (data not shown), making it unlikely that C-reserves limited N_2_ fixation. The large difference in cellular inclusion content also suggests that their subcellular metabolism influences *k*_*N*_ and *k*_*C*_. Cyanophycin comprised ∼3% of cellular N in the N_2_ culture, whereas polysaccharides comprised ∼30% of cellular C (Table 1). Moreover, the early night *y*_*N*_ values were considerably higher than the morning *p*_*C*_ values, suggesting that the turnover rate of cyanophycin is considerably faster than polysaccharide turnover at times of highest N and C assimilation, respectively. Thus, it appears that during diazotrophy, cells retain large pools of C storage with slow turnover rates and small pools of N storage with high turnover rates to manage their C and N demands. This strategy could result in a greater range of enrichment in cyanophycin inclusions compared to the larger and less dynamic pool of polysaccharides. A high turnover rate of cyanophycin also helps explain why variation in *k*_*N*_ values in NO_3_-grown cells was higher in the morning, when the cells assimilated N into cyanophycin, and lower in the afternoon, when the cyanophycin pool was bypassed. Differences in the turnover rates of N-rich proteins might also explain the large variation in *k*_*N*_ in NO_3_-grown cells during the night when N assimilation again bypassed cyanophycin.

Differences in the timing of N_2_ fixation revealed a surprising amount of within-population cell-to-cell heterogeneity in diazotrophic cultures. While the majority of cells fixed N_2_ at night as expected (Mitsui et al., 1986; Gallon, 1992; Tuit et al., 2004; Wilson et al., 2017), subpopulations in both the turbidostat-grown and semicontinuous batch cultures fixed N_2_ in the morning. Asynchronous diazotrophy has been suggested to occur when the amount of N_2_ fixed at night is insufficient to support growth in the following day (Dron et al., 2013; Rabouille et al., 2014; Rabouille and Claquin, 2016). In *Cyanothece* 51142, asynchronous diazotrophy coincided with the diel maxima in population-level C fixation (Figure 2), but single-cell analysis revealed that morning N_2_ fixation was limited to cells whose C fixation rates, and thus presumably intracellular O_2_ concentrations, were low compared to the rest of the population. This behavior may be associated with prolonged deactivation of PSII through the early morning hours (Rabouille and Claquin, 2016). How these activities are regulated is not yet known.

Semi-continuous, fed-batch cultures are exposed to a wider range of nutrient and light concentrations compared to turbidostat cultures. These variations could result in a greater range of cell physiologies within a population. Together with previous reports of N_2_ fixation in UCYN occurring during a subjective dark phase under continuous light (Colón-López and Sherman, 1998; Pennebaker et al., 2010; Dron et al., 2013), our findings suggest that the timing of N_2_ fixation is not only regulated by the circadian rhythm or light/dark cycle but also by the cell’s ability to balance N and light energy demands. The greater range of heterogeneity within the SC-N_2_ culture compared to the N_2_ culture is also consistent with the idea that cell-to-cell metabolic heterogeneity facilitates rapid population adjustment to environmental changes (Ackermann, 2015; Schreiber et al., 2016) such as those present in coastal environments (Rippka, 1988).

## Conclusion

Dual labeling combined with nanoSIMS imaging enabled a much richer and more complex view of cell activities than previously observed using measurements of bulk activities. Specifically, we observed significant cell-to-cell variation, which we attribute to differences in (1) the degree to which internal storage compounds are used as sources of C and N for cyanophycin synthesis, (2) the turnover rates of different storage pools, (3) the range of environmental conditions experienced by a population over a day–night cycle, and (4) the timing of N_2_ fixation. The intercellular heterogeneity potentially reflects adaptive mechanisms that allow *Cyanothece* to thrive in dynamic environments.

Additional details of C and N metabolism were also elucidated by evaluation of ^13^C and ^15^N labeling patterns across the day–night cycle. Cyanophycin synthesis is a highly effective N-scavenging pathway that assimilates N from protein degradation as well as external sources (NO_3_ or N_2_). Whether growing on N_2_ or NO_3_, cells prioritize cyanophycin synthesis when N assimilation rates are highest. In NO_3_-growing cells, N assimilation switches from cyanophycin synthesis to RubisCO synthesis, suggesting that there is a cyanophycin requirement that, once met, can be bypassed in favor of protein synthesis. In NO_3_-grown cells, night-time CO_2_ was assimilated into polysaccharides simultaneous with catabolism of polysaccharides used for protein synthesis, suggesting that one way these cells control C is to maintain a directional flow of new carbon entering the cell: CO_2_ → gluconeogenesis → polysaccharides → glycolysis → protein.

## Data Availability Statement

The original contributions presented in the study are included in the article/Supplementary Material, further inquiries can be directed to the corresponding author.

## Author Contributions

OP organized the experimental part of this study conducted during the 10th Group for Aquatic Productivity (GAP) workshop in August 2017. LP, TM, ME, and KH designed the study. TM, ME, and LP performed the SIP experiment. MK and LP performed the nanoSIMS analysis. MV performed the TEM analysis. LP, KH, ME, and TM drafted the manuscript, and all authors provided input during writing of the manuscript. All authors contributed to sampling and data interpretation.

## Conflict of Interest

The authors declare that the research was conducted in the absence of any commercial or financial relationships that could be construed as a potential conflict of interest.
