# Supplementary material for: Temporal Patterns and Intra- and Inter-Cellular Variability in Carbon and Nitrogen Assimilation by the Unicellular Cyanobacterium Cyanothece sp. ATCC 51142
Source: Front Microbiol. 2021 Feb 4;12:620915. doi: 10.3389/fmicb.2021.620915 (PMC7890256; doi:10.3389/fmicb.2021.620915)
Supplement: Supplementary file 1 [file Data_Sheet_1.pdf]

Supplementary Material

1 SUPPLEMENTARY TABLES AND FIGURES

Table S1. Relevant chemical and isotopic characteristics of the incubation media used for SIP experiments.

Incubation period	Culture	DIC†		N_2 or NO_3	
		$[\mathrm{mmol\,L^{-1}}]$	$x(^{13}C)_S$	$[\mathrm{mmol\,L^{-1}}]$	$x(^{15}N)_S$
07:30–09:30 (morning)	N_2	1.4	0.23	0.3	0.06
	NO_3	1.2	0.28	22.7	0.07
	SC-N_2	1.4*	0.23	0.3	0.06
14:45–17:15 (afternoon)	N_2	0.5	0.67	0.3	0.06
	NO_3	0.4	0.86	22.7	0.07
	SC-N_2	0.3	0.99	0.3	0.06
21:45–17:15 (night+day)	N_2	0.6§	0.53	0.3	0.06
	SC-N_2	0.4§	0.74	0.3	0.06
21:45–00:45 (early night)	N_2	0.6§	0.53	0.3	0.06
02:00–07:00 (late night)	N_2	1.0§	0.32	0.3	0.06
21:45–07:00 (full night)	N_2	0.6§	0.53	0.3	0.06
	NO_3	0.5§	0.68	16.2	0.10
	SC-N_2	0.4§	0.74	0.3	0.06

† DIC concentrations $[\mathrm{mmol\,L^{-1}}]$ and ^{13}C atom fractions $[x(^{13}C)_S]$ were calculated based on DIC measurements in samples from bioreactors taken at the same time point and the known amount of added $NaH^{13}CO_3$.

* Value was assumed to be the same as in the N_2 culture.

§ Values were interpolated from DIC concentrations measured at 07:00, 09:00 and 14:00.

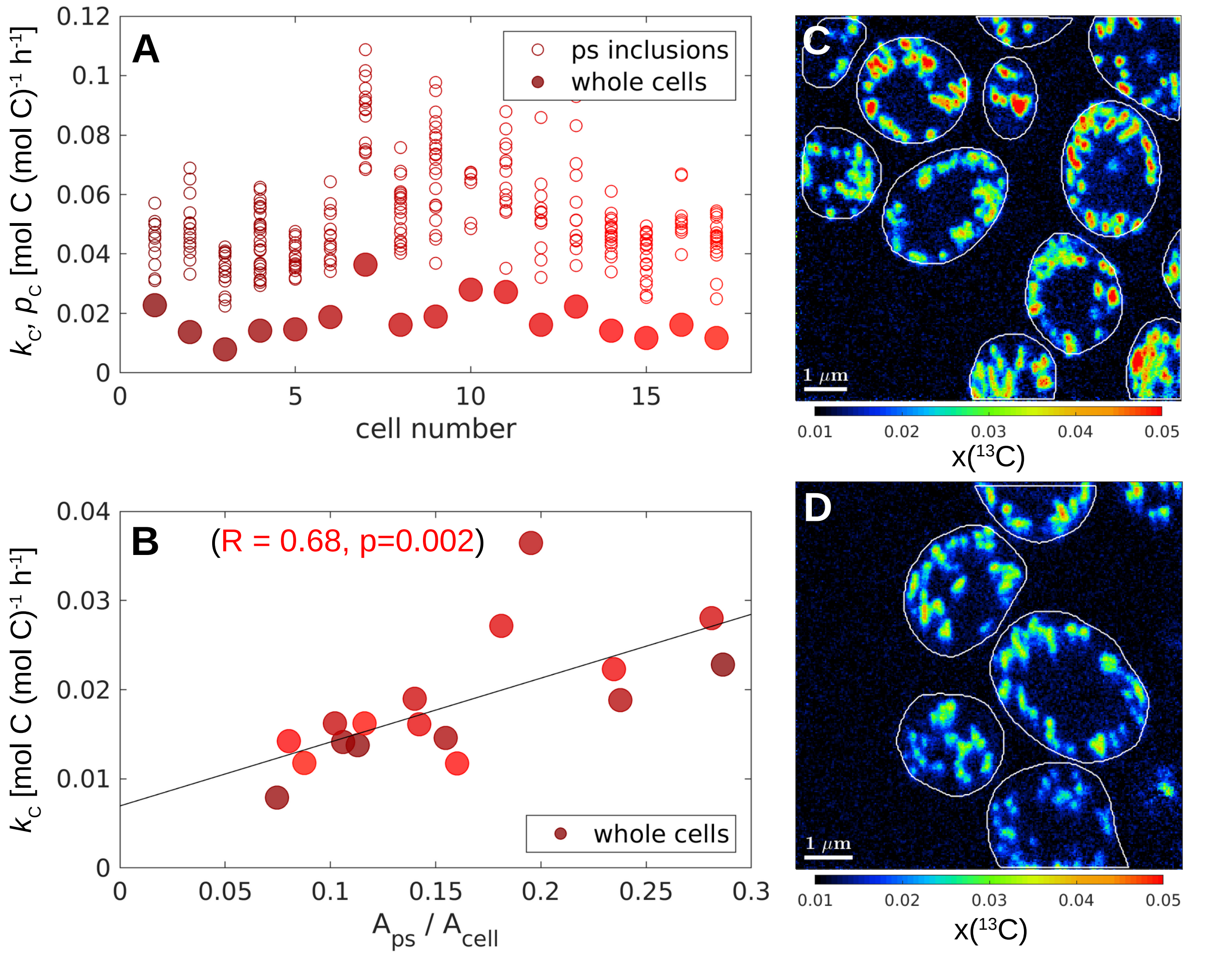

Supplementary Figure S1. Intra-cellular heterogeneity of C assimilation in N_2-fixing *Cyanothece* 51142. Shown are data for cells from the N_2 culture incubated in the morning (07:30–09:30). **(A)** Carbon-specific rates of C assimilation in individual polysaccharide (ps) inclusions (p_C; open symbols) and in whole cells (k_C; filled symbols), calculated for 279 inclusions in 17 selected cell sections. p_C varied among polysaccharide inclusions (CV $= 32\%$), with 61% and 39% of the total variance explained by differences among and within cells, respectively ($\mathrm{SS_{groups}/SS_{total}} = 0.0455/0.0746 = 0.61$, $\mathrm{SS_{error}/SS_{total}} = 0.0291/0.0746 = 0.39$). Variation in p_C values among cells was significant (ANOVA, $F(16, 262) = 25.6$, $p = 10^{-44}$). **(B)** Correlation between k_C in whole cells and the relative area of the cell sections covered by polysaccharide inclusions, $A_{\mathrm{ps}}/A_{\mathrm{cell}}$ (coefficient of determination $R^2 = 0.46$). The correlation reveals that the cell-specific ^{13}C enrichment depends on the cell volume probed by nanoSIMS. **(C–D)** Examples of ^{13}C atom fraction images from which data in panels A–B were calculated. White lines show approximate cell outlines, ^{13}C-enriched spots correspond to polysaccharide inclusions.

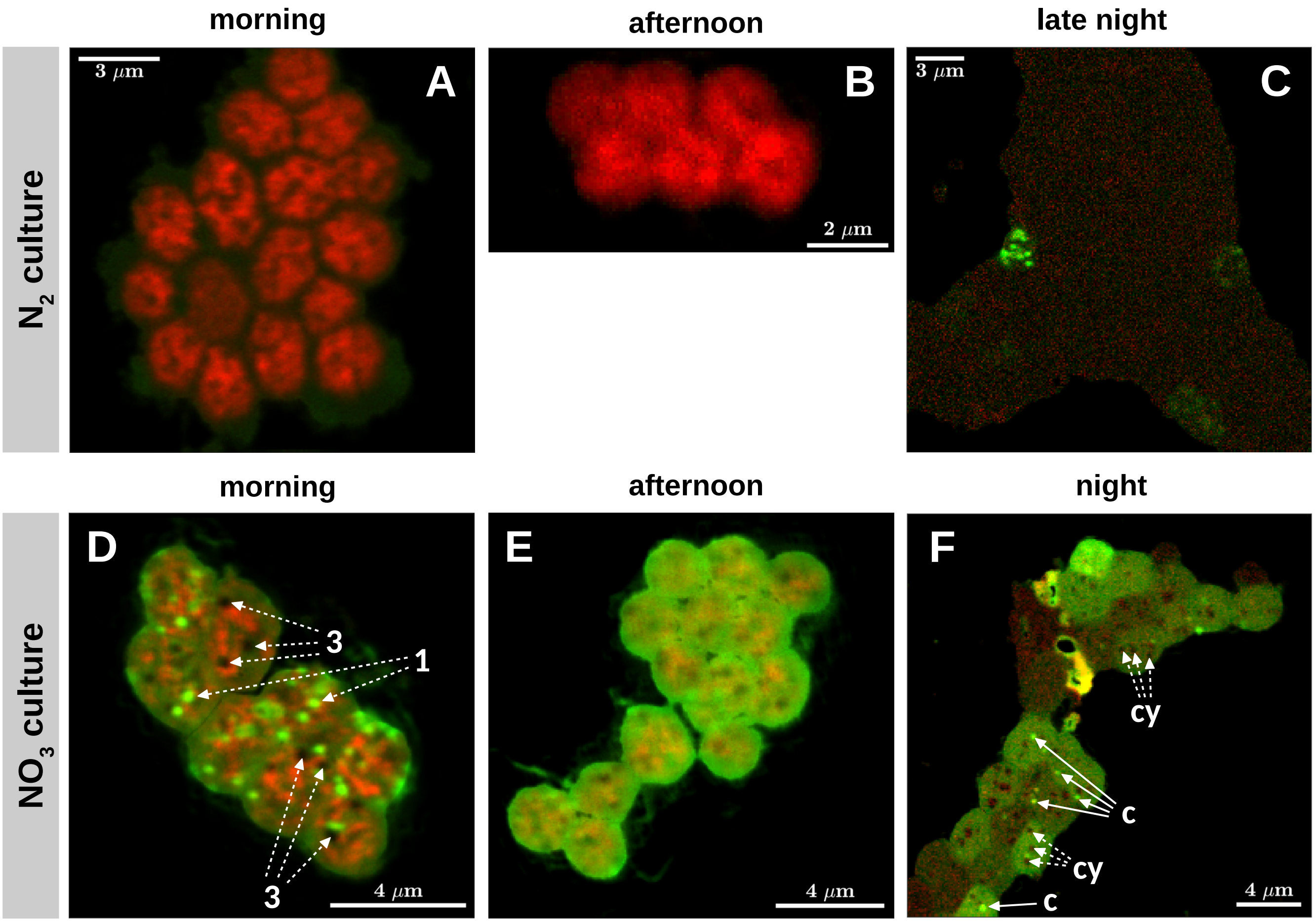

Supplementary Figure S2. Images of the isotopic composition of *Cyanothece* 51142 cells. Shown are additional overlays of the ^{13}C (red) and ^{15}N (green) atom fractions measured in cells from the N_2 and NO_3 culture deposited on polycarbonate membrane filters. Note that panel C shows cells from the N_2 culture incubated during late night (02:00–07:00). In panels D and F, examples of cyanophycin inclusions (cy) and carboxysomes (c) are marked with dashed-line and solid-line arrows, respectively.
